# Tinnitus in elderly patients and prognosis of mild-to-moderate congestive heart failure: a cross-sectional study with a long-term extension of the clinical follow-up

**DOI:** 10.1186/1741-7015-9-80

**Published:** 2011-06-29

**Authors:** Claudio Borghi, Eugenio R Cosentino, Elisa R Rinaldi, Cristina Brandolini, Maria C Rimondi, Maddalena Veronesi, Arrigo FG Cicero, Ada Dormi, Antonio Pirodda

**Affiliations:** 1 Department of Internal Medicine, Aging and Kidney Diseases, Internal Medicine Unit, Bologna, Italy; 2 Department of Surgical Sciences and Anesthesiology, Audiology Unit, Bologna, Italy

## Abstract

**Background:**

The complex mechanism responsible for tinnitus, a symptom highly prevalent in elderly patients, could involve an impaired control of the microcirculation of the inner ear, particularly in patients with poor blood pressure control and impaired left ventricular (LV) function.

**Methods:**

In order to define the relationship between the presence of tinnitus and the severity and clinical prognosis of mild-to-moderate chronic heart failure (CHF) in a large population of elderly patients (N = 958), a cross-sectional study was conducted with a long-term extension of the clinical follow-up. Blood pressure, echocardiographic parameters, brain natriuretic peptide (BNP), hospitalization, and mortality for CHF were measured. Multivariate logistic regression analysis was used to assess the association between the presence of tinnitus and some of the prognostic determinants of heart failure.

**Results:**

The presence of tinnitus was ascertained in 233 patients (24.3%; mean age 74.9 ± 6 years) and was associated with reduced systolic and diastolic blood pressure (123.1 ± 16/67.8 ± 9 vs 125.9 ± 15/69.7 ± 9; *P *= .027/*P *= .006), reduced LV ejection fraction (LVEF%; 43.6 ± 15 vs 47.9 ± 14%, *P *= .001), and increased BNP plasma levels (413.1 ± 480 vs 286.2 ± 357, *P *= .013) in comparison to patients without symptoms. The distribution of CHF functional class was shifted toward a greater severity of the disease in patients with tinnitus. Combined one-year mortality and hospitalization for CHF (events/year) was 1.43 ± 0.2 in patients with tinnitus and 0.83 ± 0.1 in patients without tinnitus, with an adjusted hazard ratio (HR) of 0.61 (95% confidence interval (CI): 0.37 to 0.93, *P *<.002).

**Conclusions:**

Our preliminary data indirectly support the hypothesis that tinnitus is associated with a worse CHF control in elderly patients and can have some important clinical implications for the early identification of patients who deserve a more aggressive management of CHF.

## Background

Tinnitus is the perception of a sound that cannot be attributed to an external source. It is a nonspecific symptom generally referable to a largely unknown dysfunction of the hearing system. A comprehensive definition has been proposed [1] to differentiate normal ear noises from pathological tinnitus defined as a head noise lasting at least five minutes and that occurs more than once per week. A distinction can also be made between subjective and objective tinnitus [2]. The former is more common and refers to an individual sound that is perceived only by the patient. From the epidemiological point of view, tinnitus affects a remarkable number of adults and is frequently associated with a hearing loss of various degrees as expression of a cochlear disorder [3,4]. In the United Kingdom approximately 4.7 million of patients are affected by tinnitus [5] and about 5% of them have experienced a severe and persistent disorder that affects their quality of life [6]. The American Tinnitus Association has reported a prevalence of about 19% (37 to 40 million), which increases with age and the degree of hearing impairment [4]. The prevalence of tinnitus has been reported to be higher in men than in women, and this difference might be related to higher hearing thresholds in the male population [3,4]. Interestingly, only 1% of patients under 45 years of age experience tinnitus, while the prevalence is about 12% in those 60 to 69 years of age and 25 to 30% in those who are > 70. Similar data recently have also been reported in a large cross-sectional study carried out with participants in the 1999 to 2004 US National Health and Nutrition Examination Surveys [7]. Several anatomical regions could contribute to the generation of tinnitus [8], even if a causative relationship between neurophysiological functions and tinnitus generation has not yet been demonstrated [9]. Moreover, several pathophysiological hypotheses have recently been proposed to explain the genesis of different kinds of tinnitus: from genetic to iatrogenic, from neurological to vascular [10]. However, a final and unique explanation is not actually available. In this complex scenario, tinnitus associated clinical conditions, such as vascular diseases, middle ear diseases, diabetes, hypertension, autoimmune disorders, and degenerative neural disorders with or without concomitant hearing loss [9-12], a functional component leading to an impaired regulation of the peripheral vascular tone can be demonstrated. For that reason, at least partly, tinnitus could be the expression of a circulatory impairment of the microcirculation of the inner ear resulting from a detrimental feedback loop between the control of systemic blood pressure and the reflex activation of the neurohumoral system (for example, sympathetic nervous system and renin-angiotensin-aldosterone system (RAAS)) [13,14]. Accordingly, any clinical condition leading to a reduction in systemic and/or regional blood flow at the ear level can trigger the onset of tinnitus or promote its exacerbation in patients already affected by this disorder.

Chronic heart failure (CHF) could be an ideal biological model to test the vascular disregulatory hypothesis of tinnitus since it is often associated with a reduced cardiac output, as well as with a reflex activation of vasoconstrictive systems, including the sympathetic nervous system and RAAS [15]. The prevalence of CHF is significantly increased in the elderly population [16], who also have a higher rate of tinnitus [3,4] and afford researchers with a reliable clinical setting to investigate the circulatory origin of hearing disorders.

The main objective of the present study is two-fold: To assess the prevalence of tinnitus in a large population of elderly patients with CHF, and to define the relationship between the presence of such an ear disorder and the severity and clinical prognosis of CHF

## Methods

This is a cross-sectional study with a long-term extension of the clinical follow-up. The researchers evaluated 958 consecutive elderly patients (age > 65 years) with a diagnosis of CHF (New York Heart Association (NYHA) functional class I to III) who were attending the Outpatients Clinic for Congestive Heart Failure of the Department of Medicine at the University of Bologna. CHF was diagnosed by the same two well-trained physicians according to the standard criteria proposed by the European Society of Cardiology [16].

Exclusion criteria were: stage 3 to 5 chronic renal failure, hypoproteinemia, recent increase in diuretic dosage (less than 30 days) or diuretic dosage change during the follow-up, concomitant use of other ototoxic drugs (that is, aminoglycosidics), continuous use of fully dosed non-steroideal antinflammatory drugs (NSAIDs). NSAID use was considered as acceptable if limited to less than one dose per week and with a limited dosage (that is, paracetamol 1,000 mg, acetilsalycilate 500 mg).

All patients underwent physical examinations and blood pressure measurements. Blood pressure was measured in the supine position after five minutes of rest, with a mercury sphygmomanometer. The systolic and diastolic blood pressure values were read to the nearest 2 mmHg. Three measurements were taken at two-minute intervals, and the average value was used to define clinical systolic and diastolic blood pressure. The presence of tinnitus was assessed by a standardized questionnaire (see Additional file 1) that allowed the categorical definition of the presence or absence of the disease based on few and specific items. The questionnaire has been largely validated in clinical practice and its sensitivity and specificity is over 80% [17-19]. All patients underwent a clinical otological and audiological examination to rule out identifiable causes of tinnitus. During the initial visit, demographic and anthropometric parameters of each patient were recorded, as well as the family history of cardiovascular disease and lifestyle information. Information was also collected about the distribution of all major cardiovascular risk factors and pharmacological treatments. The severity of CHF was defined according to NYHA functional classification, while a determination of plasma levels of brain natriuretic peptide (BNP) and a comprehensive echocardiographic evaluation, including left ventricular ejection fraction (LVEF) and end-systolic and end-diastolic volumes, were individually carried out to define more accurately the characteristics of the disease as well as the level of hemodynamic deterioration. To further define the clinical impact of tinnitus, each patient was actively followed-up on an intention-to-treat basis for a cumulative period of 12 months after enrolment in the study to assess mortality and the rate of hospitalization for CHF. The results were then compared between patients with and without hearing disorders.

The study was fully conducted in accordance with the Declaration of Helsinki. The study protocol was approved by the Ethical Committee of the University of Bologna and informed consent was obtained from all patients before the inclusion in the study.

### Statistical analysis

Results are expressed as mean ± standard deviation (SD). Normality of the distribution of the data was examined with the Kolmogorov-Smirnov test. Continuous variables are expressed as mean ± SD or median with interquartile range as appropriate, and categorical variables are expressed as a percentage. Data that were not normally distributed were logarithmically transformed before analysis. Comparisons among groups were made by *t*-test for continuous variable and chi-square tests (χ^2^) for categorical variables. Associations between variables were tested using Pearson's coefficient and correlation in the overall population of patients, as well as in patients with and without tinnitus. Multivariate logistic regression analysis was used to assess the association between the presence of tinnitus and some of the prognostic determinants of heart failure, including LVEF%, levels of BNP, age, and gender and presence of presbyacusis (bilateral loss > 30 decibels between 4,000 and 8,000 Hz). The statistical analysis was carried out by using a SPSS statistical package (version 9.6.2 for Windows; SPSS Inc., Chicago, IL, USA).

## Results and discussion

**Figure 1:**
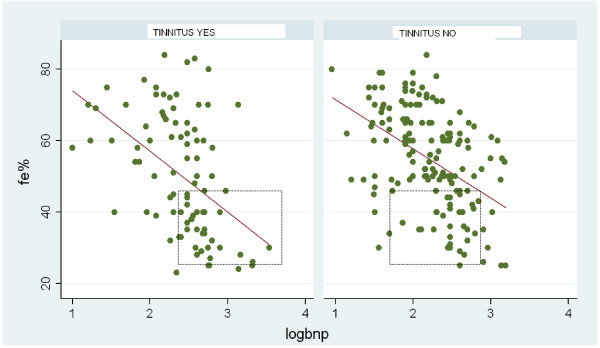
**Correlation between ejection fraction (EF%) and brain natriuretic peptide (BNP) in the group with tinnitus and those without tinnitus**.

Among the 958 patients, 530 (55.3%) were male and 428 (44.7%) female (mean age 73.9 ± 11). The presence of tinnitus was ascertained in 233 patients (24.3%; female/male: 111/122, mean age 74.9 ± 6 years), while 725 patients (75.7%; female/male: 317/408, mean age 73.6 ± 7 years) were symptom-free. The baseline clinical, echocardiographic, and neurohumoral characteristics of the population are reported in Table 1. There was no observation of any difference in the age and gender distribution between patients with and without tinnitus. Systolic and diastolic blood pressure values were significantly lower in patients with tinnitus as opposed to those without it, while the prevalence of systolic blood pressure values < 100 mmHg was 9.9% in patients with tinnitus and 5.1% in control patients. In terms of LV function, patients affected by tinnitus showed significantly lower values of EF% associated with higher plasma levels of BNP. As expected, in the overall population a linear correlation was found between LVEF% and the plasma levels of log-BNP (R = 0.413, *P *<.0001). The correlations between EF% and plasma BNP levels in patients with and without tinnitus are reported in Figure 1. A strong linear inverse relationship was observed in both populations of patients where it achieved statistical significance (patients with tinnitus: R = 0.4193, *P *<.005; patients without tinnitus: R = 0.4060, *P *<.001). A combination of reduced LVEF (< 45%) and BNP levels above the normal value (100 pg/mL) was observed in 31.3% (73/233) of the patients with tinnitus and in only 18.3% (133/725) of those free from symptoms. The difference achieves statistical significance (*P *<.003). This suggests a greater degree of systolic dysfunction in patients with tinnitus.

**Table 1:** Baseline clinical, echocardiographical, and neurohumoral characteristics of the population

	Presence of tinnitus		*P-*value
	
	Yes	No	
Male	122 (52.3%)	408 (52.6%)	NS
Female	111 (47.7%)	317 (47.4%)	NS

Ethiology of CHF (%)			
Hypertension	30.0	31.7	NS
Ischemic	41	40	NS
CMP	23.3	24.3	NS
Valve disease	3.7	3	NS
Other	2.0	1	NS

Age (years)	74.9 ± 6	73.6 ± 7	n.s.

BMI (kg/m²)	26.6 ± 4	27 ± 4	n.s
Lying SBP (mmHg)	123.1 ± 16	125.9 ± 15	.027*
Standing SBP (mmHg)	118.3 ± 18	121.3 ± 17	.024*
Lying DBP (mmHg)	67.8 ± 9	69.7 ± 9	.006*
Standing DBP (mmHg)	66.7 ± 9	68.8 ± 9	.003*
BNP (pg/ml)	413.1 ± 480	286.2 ± 357	.013*
EF (%)	43.6 ± 15	47.9 ± 14	.001*
% EF ≤ 45%	50.6 ± 12	39.9 ± 12	.001*
LV TDV (mL)	144 ± 23	118 ± 24	.001*
LV TSV (mL)	53 ± 10	46 ± 9	NS

Abbreviations: BMI, body mass index; BNP, brain natriuretic peptide; CHF, congestive heart failure; CMP, cardiomiopathy; DBP, diastolic blood pressure; EF, ejection fraction; LV, left ventricular; NS, not significant; SBP, systolic blood pressure; TDV, total distribution volume.

**Figure 2:**
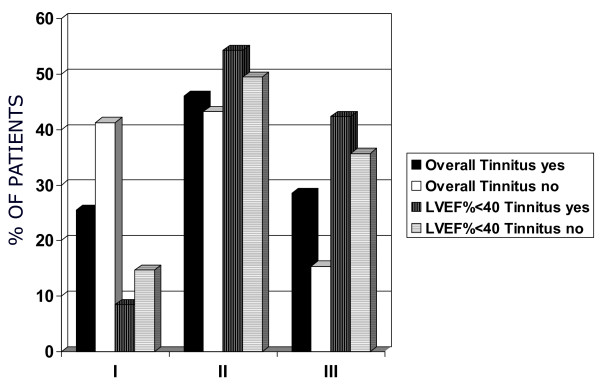
**Distribution of New York Heart Association (NYHA) functional class among all patients (*P *for trend <.001) (A) and among the subgroup with impaired left ventricular ejection fraction ((LVEF%) < 40%; *P *for trend <.05)**. **(B)**

The distribution of NYHA functional class in the population of patients with CHF and tinnitus showed a rightward shift (Figure 2) with a greater proportion of patients in NYHA classes II and III when compared with the population of patients without tinnitus. A separate analysis was carried out in patients with a more severe impairment in LVEF (< 40%), and it confirmed the greater proportion of patients in NYHA classes II and III among those affected by tinnitus (Figure 2), thereby excluding the possibility that differences in LV function are primarily responsible for the observed distribution of NYHA functional class in patients with and without tinnitus. Multivariate logistic regression analysis (Table 2) showed that the presence of tinnitus was mainly associated with reduced LVEF% (< 45%). Additional risk factors were NYHA > II to III class and higher levels of BNP, while age, gender and presence of presbyacusis were not directly related with tinnitus.

**Table 2:** Multivariate analysis of clinical correlates with the presence of tinnitus in the patients population

Variable	Odds ratio	*P-v*alue
Age > 70 years	1.02	0.14

Gender female	0.97	0.23

LVEF % < 45	1.28	0.001

BNP > 200 mg/dL	1.09	0.05

Presbyacusis yes*	1.02	0.27

*Bilateral loss of 30 decibels between 4,000 and 8,000 Hz.

The percentages of distribution of concomitant drug treatment in the two populations of patients are reported in Table 3. Patients with tinnitus showed a greater use of angiotensin receptor blockers and diuretics, which probably reflects the larger proportion of patients with more severe NYHA functional class and might explain the lower BP values. Repeating the survival analysis by angiotensin receptor blockers use, the trend was not significantly different between users and non-users. Interestingly, tinnitus was also associated with a greater use of NSAIDs, which could partially explain the differences in terms of clinical profile and LV function between patients with and without tinnitus, even if those drugs were used on demand and at low dosage. On the other side, repeating the survival analysis by NSAID use, the trend was not significantly different between users and non-users.

**Table 3:** Distribution of concomitant drug treatment

Therapy	Yes	No	Tinnitus Yes	Tinnitus No	*P-*value
ACE-inhibitors	53.24	46.76	51.50	53.79	NS
ARBs	52.92	47.08	63.95	49.38	.001
Antiplatelets	86.22	13.78	84.55	86.76	NS
NSAIDs	22.38	77.62	37.07	17.68	.001
Ca antagonist	19.83	80.17	16.81	18.89	NS
Warfarin	19.24	80.76	20.35	56.55	NS
Diuretics	58.77	41.23	65.67	53.9	.014
Alfa-blockers	5.45	94.5	5.60	4.66	NS
Beta-blockers	42.48	57.52	75.06	78.2	NS
Statins	5.38	94.62	7.82	4.63	NS
Nitroderivates	25.45	74.55	22.84	26.28	NS
Antialdosterone drugs	14.03	85.97	16.81	13.14	NS

Abbreviations: ACE, angiotensin-converting enzyme; ARB, angiotensin receptor blocker; Ca, calcium; NS, not significant; NSAIDs, nonsteroidal anti-inflammatory drugs.

**Figure 3:**
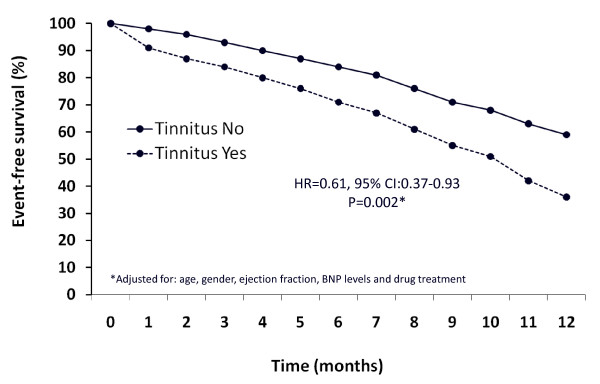
**Twelve-month event-free survival in congestive heart failure (CHF) patients with or without tinnitus**.

In terms of clinical prognosis, the combined rate of death plus hospitalization for CHF (events/year) during the 12 months after the enrolment in the study was 1.43 ± 0.2 in patients with tinnitus and 0.83 ± 0.1 in patients without tinnitus, with a Hazard Ratio of 0.61 (95% CI: 0.37 to 0.93). The difference achieved statistical significance (*P *<.002) and persisted after adjustment for major confounding prognostic factors (age, gender, EF, BNP levels, and drug treatment; Figure 3).

Tinnitus affects a considerable number of adult patients, and its prevalence increases with age, with a large proportion of the elderly population affected by this common hearing disorder. The results of the present study suggest that in elderly patients with CHF, the presence of tinnitus is associated with a higher prevalence of LV dysfunction. Moreover, a large proportion of patients with tinnitus showed the combination of low levels of EF with elevated plasma levels of BNP as a measure of systolic dysfunction and cardiac overload. The one-year mortality and hospitalization for CHF was significantly increased in patients with tinnitus as an expression of a greater degree of clinical instability of the disease. From a speculative point of view, this suggests the possibility that the onset of tinnitus or the worsening in its severity may be considered as an indirect symptom of poor hemodynamic stability in elderly patients with CHF. Of course, the causal relationship between CHF and tinnitus onset or worsening has to be further investigated with more appropriate studies. In fact, tinnitus could have origins other than or concomitant to circulatory disorders, including localized cochlear disorders [20], hormonal causes [21], dysregulation of the limbic system [22] or a complex interplayamong sensory, cognitive and affective systems [23]. However, the mechanism responsible for the increased rate or the exacerbations of tinnitus in elderly patients with CHF could also be identified in the combination between the hemodynamic failure typical of CHF and the lack of any effective autoregulatory control of inner ear circulation leading to a sudden or persistent systemic blood pressure decrease. The possibility that the onset of tinnitus is related to a systemic hypotensive response involving directly or indirectly the microcirculation of the inner ear has already been investigated in patients with and without hypertension [24-26]. An increase in the prevalence of tinnitus has been found in patients with hypertension aggressively treated with blood pressure lowering drugs, while an interesting relationship has been described between the time-course of individual hypotensive episodes and the onset of hearing symptoms [24]. In the present study, blood pressure values were lower in the advanced NYHA classes (NYHA I to II 127.5 ± 13/70.2 ± 7 vs NYHA III 119.4 ± 11/65.1 ± 7; *P *<.0001) as a consequence of the greater impairment of LV function. This could largely explain the differences in the onset of tinnitus in the different subsets of patients with CHF. The same explanation applies to the correlations between the prevalence of tinnitus and the levels of LVEF% or BNP, suggesting a close relationship between the systemic hemodynamic profile and the response of the inner ear circulation and/or the ear sensorineural pathway. This would reasonably exclude the possibility of an exclusive role of a primary sensorineural derangement in patients with CHF, since if this was the case the prevalence of tinnitus would be largely independent from the extent of LV dysfunction. In fact, the decrease in cardiac output and the associated vasoconstrictive response observed in patients with heart failure can significantly reduce the blood supply to the inner ear. This peripheral hemodynamic response cannot be compensated by a modification of arterial tone because of the peculiar characteristics of the inner ear circulation [11,12] and would result in the development of a local condition of hypoperfusion/ischemia with the activation or enhancement of the sensorineural mechanisms that produce tinnitus. The degree of the ischemic response would be proportional to the decrease in blood supply, as well as to the extent of functional heart failure; thus supporting the relationship observed between severity of CHF and percentage of patients affected by tinnitus. The close relationship between impaired LV function and presence of tinnitus is also supported by the observation of a worst clinical prognosis in the population who complain about the symptoms. As an alternative hypothesis, the onset of tinnitus can be related to the high prevalence of depressive symptoms in patients with CHF. We estimated the rate of depression in our population by DSM-III criteria and the proportion of affected patients was comparable between those with and without tinnitus (47% vs. 44%; *P *= NS). This reasonably excludes a primary role for depression in the onset or exacerbation of tinnitus, while the importance of such a disease as responsible for these symptoms in the overall population cannot be ruled out. On the other side, the lack of association between tinnitus and auditory thresholds in our patients could be related to the extreme variability in the entity of the hemodynamic derangement that affect the patients with heart failure and that could promote the onset of tinnitus on an ischemic and reversible basis without any major evidence of a concomitant and persistent hearing impairment. Actually, tinnitus is a spontaneous symptom that is immediately perceived without any external stimulation, whereas the perception of hearing impairment needs an external acoustic trigger. This could have increased the sensitivity of our patients toward tinnitus beyond the complaint of any hearing loss. We could also speculate about the possibility that two separate mechanisms of action might contribute to the same symptom, namely one functional (hemodynamic derangement) and the other more structural (hearing disturbance).

The clinical relevance of tinnitus is primarily dependent on the degree of LV dysfunction--the major responsibility for signs and symptoms of heart failure. However, the results of the logistic regression analysis support an independent role for the presence of tinnitus after adjustment for the major confounding risk factors, including any measure of LV dysfunction. The present study probably provides the first evidence of the possibility that the hearing system can be involved in the overall assessment of the clinical outcome of patients with CHF in addition to typical prognostic indicators (for example, LVEF%, plasma levels of BNP).

The use of tinnitus as a measure of severity and stability of heart failure in the elderly population could be complementary to the use of hemodynamic or biochemical markers, since it is easy to assess, does not require any blood sample or instrumental evaluation, and can be perceived by patients when other signs or symptoms of the disease have not yet developed. In particular, the observation of an increase in the relative risk of major cardiovascular events in patients with CHF and tinnitus might support the possibility that the onset of tinnitus can be considered as a useful indicator of a decline in cardiac performance well before the development of the typical manifestations of heart failure. This is supported by the higher proportion of patients treated with NSAIDs among those who complain of tinnitus (see Table 2). Indeed, the use of NSAIDs is one of the most common causes of hospitalization in patients with chronic CHF [27]. This is because the use of such drugs can impair renal function and promote fluid overload with a resulting decline in LV systolic function, which leads to the onset of tinnitus, as well as to symptoms of heart failure.

As expected, the study has several limitations according to its design and the nature of the observation. First, the study was carried out exclusively in the elderly population and it is unknown if the presence of tinnitus has the same relevance in younger patients with CHF. Nevertheless, since the relationship between tinnitus and CHF appears to be mainly related to the extent of decline in LV function, we should expect a unique causative mechanism regardless of the age of the patients. Second, the enrolment of patients was based on the use of an individual questionnaire without any additional and confirmatory evaluation of the presence of tinnitus. According to the universally accepted definition, tinnitus is a perceived sound that cannot be attributed to an external source. This implies only a categorical estimate based on its presence or absence. In addition, the questionnaire provides a semiquantitative estimate of the presence of tinnitus and has been validated in a large population of patients with a different set of comorbidities where it has been demonstrated to be accurate and reproducible for the diagnosis of tinnitus [17-19]. Third, no information was provided about the relationship between changes in LV function and the onset and persistence of tinnitus over a period of follow-up. However, the study protocol was finalized to assess if the presence of tinnitus at any time in the clinical history of the patients might affect the long-term prognosis. The discriminatory power of this approach could have been biased by those patients whose tinnitus had developed after the baseline evaluation, even if the difference observed in terms of mortality and morbidity between the two groups of patients did not support such interpretation. Finally, the study does not provide any information about the meaning of tinnitus in patients with cardiovascular diseases other than CHF. It is unknown if the presence of tinnitus is the nonspecific consequence of a reduction in regional blood flow at the level of the inner ear or if it necessarily implies a concomitant activation of the sympathetic nervous system and RAAS as occurs in most patients with CHF. The evidence provided by the studies carried out in patients with hypotension supports a generalized hemodynamic mechanism, but the importance of the involvement of the neurohumoral system cannot be ruled out and deserves further investigations.

In our patients, microcircular dysfunction of the inner ear may generate tinnitus without having an influence on auditory thresholds

## Conclusion

To date, this is the first large, cross-sectional, clinical study supporting an association between tinnitus and CHF control in elderly patients. Data suggest that the onset of tinnitus might be affected by the degree of decline in LV function and is probably the consequence of an insufficient autoregulatory mechanism at the level of the circulation of the inner ear. These data can have some important clinical implications including the possibility that the onset and/or worsening of tinnitus can antedate the destabilization of CHF. This would allow for the early identification of patients who deserve a more aggressive management of heart failure or an adjustment of drug treatment, including a cautious administration of NSAIDs. If confirmed by larger prospective studies, this evidence would indirectly contribute to improve the quality of life of patients with CHF and might reduce the rate of hospitalization, as well as the huge economic burden of the management of CHF.

## Abbreviations

BNP: brain natriuretic peptide; CHF: chronic heart failure (or congestive heart failure; EF: ejection fraction; LV: left ventricular; NSAIDs: non-steroideal antinflammatory drugs; NYHA: New York Heart Association; RAAS: renin-angiotensin-aldosterone system; SD: standard deviation

## Competing interests

No author has any direct or indirect conflict of interests in the publication of this article. In particular, the study was observational and has no direct relevance for drug prescription.

## Authors' contributions

CB and AP had a main role in study concept and design, moreover they respectively coordinated the internal medicine and the ORL staff. ERC, ERR and MV mainly enrolled the patients and followed-up in regard to heart failure symptomology and therapy. CB and MCR executed the ORL screening. AD and AFGC mainly worked on data analysis and interpretation. All authors read and approved the final article.

## Pre-publication history

The pre-publication history for this paper can be accessed here:

http://www.biomedcentral.com/1741-7015/9/80/prepub

## Supplementary Material

Additional files 1 **Questionnaire sent to asses the presence of tinnitus**. Click here for file
