# Supplementary material for: Tinnitus in elderly patients and prognosis of mild-to-moderate congestive heart failure: a cross-sectional study with a long-term extension of the clinical follow-up
Source: BMC Med. 2011 Jun 29;9:80. doi: 10.1186/1741-7015-9-80 (PMC3141544; doi:10.1186/1741-7015-9-80)
Supplement: Additional files 1 — Questionnaire sent to asses the presence of tinnitus. [file 1741-7015-9-80-S1.DOC]

**Additional file 1**

We would like to ask you about your assessment of your hearing system. With just a few questions, we will be able to identify whether or not you have some problems in the way you hear or in the way you feel comfortable with your hearing system.

Just answer “yes” or “no” to the following questions (put a “X” in the space).

|  | **Yes** | **No** |
| --- | --- | --- |
| 1. Do you think that there is “something wrong” with your hearing? |  |  |
| 1. Do you hear any noise, whistle, or any other disturbing sensation inside your ears? |  |  |

If you have answered “yes” to either question 1 or 2, please continue with the following set of questions:

|  | **Yes** | **No** |
| --- | --- | --- |
| 1. Do your hearing problems affect just one side? |  |  |
| 1. If yes, it is always the same side? |  |  |
| 1. Do you hear the noise only in one side? |  |  |
| 1. If yes, it is always the same side? |  |  |
| 1. Does the noise change its characteristics? |  |  |
| 1. Do you feel the noise all year long? |  |  |
| 1. Do you feel the noise all day long? |  |  |
| 1. If not, do you hear the noise mainly during the night? |  |  |

Regardless of your previous answers, please provide an answer to the very last question.

|  | **Yes** | | **No** |
| --- | --- | --- | --- |
| 1. Do you know if you have suffered or may be affected by ear diseases? |  | |  |
|  | **Yes** | **No** | |
| **Total (count of yes/no)** |  |  | |

**Diagnosis of tinnitus**

If the number of yes was >6/10 and the answer to question 11 is no.

If the number of yes is >8/10 and the answer to question 11 is yes.
